# Impairment of Angiogenic Sphingosine Kinase-1/Sphingosine-1-Phosphate Receptors Pathway in Preeclampsia

**DOI:** 10.1371/journal.pone.0157221

**Published:** 2016-06-10

**Authors:** Aneta Dobierzewska, Macarena Palominos, Marianela Sanchez, Michael Dyhr, Katja Helgert, Pia Venegas-Araneda, Stephen Tong, Sebastian E. Illanes

**Affiliations:** 1 Department of Obstetrics & Gynecology and Laboratory of Reproductive Biology, Faculty of Medicine, Universidad de los Andes, Santiago, Chile; 2 Translational Obstetrics Group, Department of Obstetrics and Gynecology, University of Melbourne, Mercy Hospital for Women, Heidelberg, Victoria, Australia; 3 Department of Maternal-Fetal Medicine, Clinica Davila, Santiago, Chile; VU University Medical Center, NETHERLANDS

## Abstract

Preeclampsia (PE), is a serious pregnancy disorder characterized in the early gestation by shallow trophoblast invasion, impaired placental neo-angiogenesis, placental hypoxia and ischemia, which leads to maternal and fetal morbidity and mortality. Here we hypothesized that angiogenic sphingosine kinase-1 (SPHK1)/sphingosine-1-phosphate (S1P) receptors pathway is impaired in PE. We found that *SPHK1* mRNA and protein expression are down-regulated in term placentae and term chorionic villous explants from patients with PE or severe PE (PES), compared with controls. Moreover, mRNA expression of angiogenic *S1PR1* and *S1PR3* receptors were decreased in placental samples of PE and PES patients, whereas anti-angiogenic *S1PR2* was up-regulated in chorionic villous tissue of PES subjects, pointing to its potential atherogenic and inflammatory properties. Furthermore, in *in vitro* (JAR cells) and *ex vivo* (chorionic villous explants) models of placental hypoxia, *SPHK1* mRNA and protein were strongly up-regulated under low oxygen tension (1% 0_2_). In contrast, there was no change in SPHK1 expression under the conditions of placental physiological hypoxia (8% 0_2_). In both models, nuclear protein levels of HIF1A were increased at 1% 0_2_ during the time course, but there was no up-regulation at 8% 0_2_, suggesting that SPHK1 and HIF1A might be the part of the same canonical pathway during hypoxia and that both contribute to placental neovascularization during early gestation. Taken together, this study suggest the SPHK1 pathway may play a role in the human early placentation process and may be involved in the pathogenesis of PE.

## Introduction

PE affects 5–8% of all pregnancies worldwide and is a leading cause of maternal and fetal morbidity and mortality [1]. This pregnancy disorder is defined as new onset hypertension and proteinuria or end-organ dysfunction (kidney and liver) appearing after 20 weeks of gestation in a previously normotensive woman [2–3]. PE is thought to result from an abnormal placenta, the removal of which ends the disease in most cases [4]. During normal early pregnancy, the hypoxic environment determines adequate extravillous trophoblast invasion into maternal decidua and proper placental vascularization and angiogenesis to allow normal blood flow between the mother and fetus [5–7]. In PE-complicated pregnancies, shallow trophoblast invasion and reduced uteroplacental perfusion leads to persistent placental hypoxia that results in abnormal up-regulation of HIF1A, increased concentrations of circulating/ placental anti-angiogenic factors (sFLT-1 and s-ENG) thus resulting in maternal angiogenic imbalance and development of systemic endothelial dysfunction [8, 9].

Despite ongoing research into the characterization of molecular mechanisms that finally triggers PE, its exact pathogenesis remains incompletely understood. However, recent evidence highlights the potential involvement of pro-survival and angiogenic bioactive lipid—sphingosine-1-phosphate (S1P) and its synthetizing enzyme–sphingosine kinase (SPHK) in the process of trophoblast differentiation/invasion [10, 11] and placental angiogenesis [12]. All these cellular and physiological processes are important to establish a healthy placenta, which is abnormal and compromised by persistent hypoxia and ischemia in PE patients [3].

Sphingosine kinase (two major isoforms: SPHK1, SPHK2) catalyzes the formation of sphingosine-1-phosphate (S1P) from the precursor sphingolipid—sphingosine [13, 14]. SPHK1 is found in the cytosol of eukaryotic cells, and it migrates to the plasma membrane upon activation with different stimuli, including PDGF [15], EGF [16], TNF-alpha [17], or S1P itself [18] to name few. SPHK1 is also up-regulated by low oxygen tension [19] and increased levels of SPHK1 are found in many human solid tumors [20] underlying its role in tumor neovascularization and angiogenesis [21]. It has been proposed that in cancer cells SPHK1 is a master regulator of hypoxia, that acts upstream of HIF1A and thus mediates the adaptation to a hypoxic environment [19].

Sphingosine-1-phosphate (S1P), synthetized by SPHK, is highly angiogenic and has been named the ´anti-apoptotic metabolite´ of ceramide [13]. Intracellularly, it regulates proliferation and survival, and extracellularly, through G-protein-coupled S1P receptors (S1PR1-5), it regulates the vascular development during embryogenesis, wound repair and cancer metastasis [14, 22]. S1P is a blood borne lipid mediator, found in association with lipoproteins such as HDL and with albumin [23, 24]. The major source of plasma S1P is believed to be red blood cells, vascular endothelial cells (ECs), and activated platelets [23, 25–26]. The systemic effects of S1P are mediated through its receptors. Recently it has been demonstrated that S1PR1 and S1PR2 are expressed on endothelial cells of the mesometrium [27], and S1PR1, -3, and -5 together with SPHK1 were found to be expressed in human placenta and trophoblast cells [27].

Moreover, recent data confirms the importance of SPHK/S1P pathway in the reproductive system. SPHK double knockout-mice (*sphk1-/-*, *sphk2-/+)* displays severely impaired uterine decidualization and uterine angiogenesis that leads to uterine hemorrhage and early embryonic lethality [12]. However, this knock-out mice model did not show abnormalities during early implantation process [12]. Studies of mice where the S1P receptor has been knocked out suggest that S1PR1 is crucial for vascular development, whereas S1PR2 and S1PR3 display partially redundant functions during vasculogenesis [28, 29].

Recent *in vitro* studies also demonstrate the important role of SPHK1/S1P pathway in functional and structural differentiation of villous trophoblast [10], and in up-regulation of extravillous trophoblast cell invasion via S1P/S1PR1 axis activation [30]. Also S1P/S1PR5 signaling has been shown to affect trophoblast migration and HUVEC tube formation *in vitro* by regulating the angiogenic phenotype of decidual natural killer (NK) cells [11]. The authors proposed that S1P pathway might represent a potential target for future treatment of gestational diseases such as preeclampsia that is characterized by inadequate dNK/trophoblast-coordinated uterine spiral artery remodeling [11].

In this study, we hypothesized that angiogenic SPHK1/S1P pathway is impaired in preeclampsia. First, we evaluated SPHK1 expression in *in vitro* and *ex vivo* models of placental hypoxia, to elucidate the potential role of SPHK1 in the human early placentation process. Then we evaluated *SPHK1* mRNA and protein levels in term placentae and term chorionic villous explants in patients with PE or severe PE (PES) in comparison with normotensive controls and the expression of angiogenic *S1PR1*, *S1PR2* and *S1PR3* receptors in the same samples.

## Materials and Methods

### Study design

This study was approved by Clinica Davila and the Universidad de los Andes (Santiago, Chile) Ethics Committee, and written informed consent was obtained from all study subjects for collection of placental samples.

Human placentae from pregnancies complicated by preeclampsia (PE) and their respective normotensive controls (CTL) were obtained with written informed patient consent. Clinical characteristics of patients, placentae and newborns are summarized in Table 1. Seventeen preeclamptic (PE) patients (moderate preeclampsia PEM (n = 8) and severe preeclampsia PES (n = 9)) and sixteen controls (CTL) were recruited for this study. Both groups consisted of women with singleton gestation and none of them took multivitamins and aspirin during pregnancy. Preeclampsia was diagnosed based on the presence of hypertension (arterial pressure (AP) higher or equal to AP 140/90 mmHg on two occasions separated by 6h or higher or equal to 160/110 mmHg in one occasion) that occurred after 20 weeks of gestation, and proteinuria (300mg/24h). Controls, who did not differ in racial origin from PE patients, were healthy subjects without pregnancy complications or chronic medical problems.

**Table 1:** Clinical characteristics of patient and delivery.

Variable	Normotensive Controls (CTL) n = 16	Preeclampsia Moderate (PEM) n = 8	*p* value (CTL vs PEM)	Preeclampsia Severe (PES) n = 9	*p* value (CTL vs PES)
Maternal age (years)	33.3 ± 5.3	31.0 ± 6.2	p = 0.14	29.8 ± 5.2	p = 0.062
Gestational age of delivery	39.05 ± 0.98	36.5 ± 3.4	p = 0.0045**	36.5 ± 3.75	p = 0.008*
Newborn weight (g)	3582.5 ± 837.8	3231.8 ± 429.1	p = 0.1	2809 ± 891.9	p = 0.02*
At 21–23 weeks Systolic pressure (mmHg)	121.3 ± 11.9	144.1 ± 9.8	p = 0.00001**	150 ± 12.51	p = 0.0003**
At 21–23 weeks Diastolic pressure (mmHg)	70.9 ± 9.3	96 ± 9.54	p = 0.00001**	94.91 ± 5.27	p = 0.00001**

Values are given as a mean ± (SD). Statistical significance was assessed using Student T- test.

* *p*≤ 0.05

** *p*≤ 0.005.

### Human placental tissue samples

Placental tissues were obtained within 15min of delivery from all subjects. Placental samples (~ 4 x 4 cm section) were randomly excised from the fetal-maternal interface region. The placental tissue was re-sectioned into small pieces, rinsed in ice-cold physiological saline (0.9% NaCl) to remove maternal blood contamination. For RNA analysis to preserve RNA integrity, placental samples were stored in ´RNA later solution´ (Ambion, USA) for 24h and processed further to isolate RNA. For protein analysis, placental samples were snapped frozen in liquid nitrogen and stored at -80°C until use. The RNA integrity of all placental samples was determined by standard RNA agarose denaturing gel.

### Third trimester villous explant culture

Third trimester human placentae obtained by C-section were placed on cold ice packs, delivered from Clinica Davila to Laboratory of Reproductive Biology, Universidad de los Andes and processed within 2h of collection. The placental tissue was washed in sterile PBS and aseptically dissected to remove endometrial tissue and fetal membranes. Fragments of placental villi tissue were cut in small pieces and additionally washed multiple times with sterile PBS to remove access of placental blood. Isolated placental villous trees were transferred into 75cm^2^ flasks (six trees per flask). Explants were cultured in DMEM-Ham’s F-12 (DMEM/F12; Life Technologies, Grand Island, NY) supplemented with 10% FBS and Pen/Strep, up to 24hrs to ensure the viability of the explant cultures.

### Cell Culture

The human choriocarcinoma trophoblastic cell line—JAR cells were cultured according to the protocol recommended by the American Type Culture Collection (ATCC, Manassas, VA, USA) in RPMI-1640 medium (HyClone, ThermoScientific, Logan, UT) supplemented with 10% heat-inactivated fetal bovine serum (HyClone), 2mM L-glutamine, 100 U/ml penicillin, and 100 μM streptomycin (HyClone) in 75 cm^2^ flasks at 37°C in a humidified 5% CO_2_ incubator. When the cells reached 80–90% confluence, the cells were detached by trypsinization and split at ratio 1:10.

### Hypoxic treatment

Hypoxic conditions were obtained by replacing oxygen with N_2_, using a Heracell 150i CO_2_ incubator (Thermo Scientific). To monitor the oxygen percentage in the incubator, the Multi-Gas Detector, model Drager X-am 2000 (Lubeck, Germany) was used. Briefly, 1 x 10^6^ JAR cells/ml were seeded in 10cm dishes 48 h before treatment. When the cells reached 60–70% confluence, they were grown under hypoxic conditions (1% or 8% oxygen) for 3, 6, 8, 16 and 24 h. The atmospheric content (21% oxygen) was used as a control at the same time course experiments. For experiments with chorionic villous explants isolated from human term placentae, six villous trees were placed in each 75cm^2^ bottle supplemented with DMEM (10% FBS, Pen/Strep) and exposed to hypoxia (1% or 8% O_2_) for 3 and 16h, respectively.

### Quantitative real-time PCR

Total RNA was extracted from frozen placental tissue or JAR cells using Master Pure Complete DNA and RNA purification kit (Epicentre, Madison, WI) according to the manufacturer´s instructions. Total nucleic acid extraction was followed by DNase I treatment to remove DNA contamination. The amount and purity of RNA was assessed by spectrophotometry (UV-Vis NanoDrop 2000; ThermoScientific, Rockford, IL). cDNA was prepared from total RNA (3 μg) using a reverse transcription system (random hexamers, Improm II Reverse Transcriptase system; Promega). PCR was performed on 50ng and 5ng cDNA samples per 20ul reaction in triplicate for each experiment using SYBR Green Master Mix (Roche Applied Science) and specific primers. Reactions were run and amplicons were detected on Mx3000P Real-time PCR thermocycler (Stratagene, La Jolla, CA). Specific primer sequences and gene accession numbers for qPCR for each gene are shown in Table 2. The relative mRNA abundance in each experimental condition was normalized to the level of *18S* RNA. The results of the real-time PCR analysis were calculated based on the ΔΔCt-method as described before [31].

**Table 2:** Primer sequences used for RT-qPCR.

Gene	Accession Number	Primer sequence	Primer sequence
		Sense	Antisense
h*SPHK1*	NM_182965.2	GGAGACCGCCATCCAGAA	CTCATAGCCAGCATAATGGTTCAA
h*S1PR1*	NM_001400.4	GCTCTCCGAACGCAACTT	CGATGAGTGATCCAGGCTTT
h*S1PR2*	NM_004230.3	TAGCCAGTTCTGAAAGCCCCA	GTTTCCAGCGTCTCCTTGGTA
h*S1PR3*	NM_ 005226.3	CCCATCTGGCATTCGAGCG	GTTGAAAAAGGGCTCCTCCGTC
h*18S*	NM_ 001082287	CAAGGAGCAGGTGGTGAAA	TTCAGGTCGTTCAGTGTCTTC

*SPHK1*, Sphingosine kinase-1*; S1PR1*, sphingosine-1-phosphate receptor 1, *S1PR2*, sphingosine-1-phosphate receptor 2, *S1PR3*, sphingosine-1-phosphate receptor 3

### Western blot analysis

The cytoplasmic and nuclear fractions of placental tissues (5mg), or chorionic villous explants or JAR cells (10^6^ cells) were obtained according to the manufacturer's instructions by using NE-PER Nuclear and Cytoplasmic Extraction Reagents (Thermo Scientific, Rockford, IL) supplemented with protease and phosphatase inhibitor cocktails (ThermoScientific, Rockford, IL). Total protein concentration in each fraction was measured using the BCA Protein Assay Kit (Thermo Scientific, Rockford, IL, USA). Fifty μg of cytoplasmic fractions or 25 μg of nuclear fractions were separated on 10% Tris-Glycine SDS-PAGE gels and transferred to PVDF membranes (Thermo Scientific, Rockford, IL) by wet blotting. Western blot analysis was performed according to standard conditions. The membranes were first incubated for 1h at room temperature in TBS solution containing 0.1% Tween-20 and 5% of non-fat dry milk to block nonspecific binding. After washing with 0.1% Tween-20 in 1XPBS, the specified proteins were detected by incubation of the blots (overnight, 4°C) with the following primary antibodies: rabbit anti-SPHK1 (1:500, Cell Signalling, #3297), mouse anti-HIF1A (1:1000, Abcam, ab113642), or rabbit anti-β-Actin (1:5000, Abcam, ab6276). After washing, the membranes were incubated with appropriate HRP-conjugated secondary antibody (1:5000, KPL) for 1 h at room temperature. For SPHK1 primary antibodies, the secondary Ms x Rb Light Chain specific HRP-conjugated antibodies (Millipore, MAB201P) were used. Proteins were detected using the Western Chemiluminescent HRP Substrate (ThermoScientific, Rockford, IL) and Kodak film development. The blots were scanned and densitometric analysis was performed using the Image Studio Lite program ver 3.1 (PerkinElmer, Life Sciences, Boston, MA). β-Actin was used as a loading control for JAR cell lysates, while Ponceau S staining of the membranes was used as an arbitrary loading control for placental and chorionic villi lysates as demonstrated previously [32]. For general protein stain with Ponceau, a box of identical dimension was used to integrate the signal within each sample on the membrane, and the intensities of stained protein bands were quantified using the Image Studio Lab program.

### Statistical analysis

All data are represented as mean ± SEM of at least three independent experiments from JAR cells and chorionic villous explants (+/- hypoxia), and from 16 normotensive and 17 preeclamptic placentae. Statistical significance between the groups was determined by nonparametric Mann-Whitney *U-test* (clinical data) or Student’s unpaired *t* test *(in vitro* studies in JAR cells and chorionic villous explants) using GraphPad Prism software (San Diego, CA). Data were considered statistically significant at p *value ≤ 0.05 and **p ≤ 0.005.

## Results

### SPHK1 is up-regulated by hypoxia in human trophoblastic JAR cells and normal human term placental chorionic villous explants

**Fig 1:**
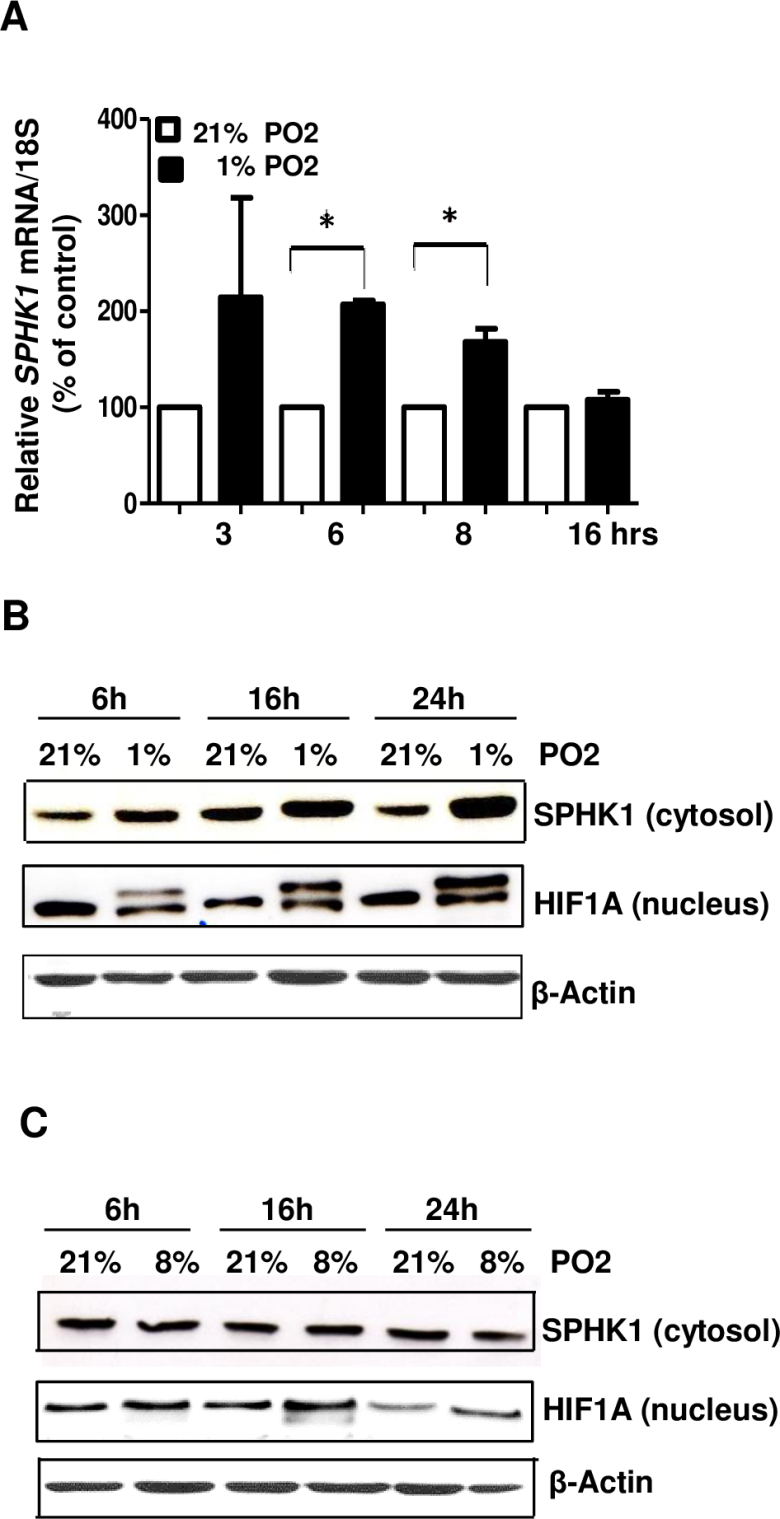
**SPHK1 is up-regulated at low oxygen tension (1% PO**_**2**_**) in human choriocarcinoma JAR cells (A)** Bar diagram shows the relative mRNA expression of *SPHK1* transcripts in JAR cells during time course (3–16h) under 21% O_2_ and 1% O_2_ exposure. Data was normalized against 18S gene. Bar graph represents mean ±S.E.M., * indicates p ≤ 0.05, n = 3. **(B, C)** Representative Western blots of cytosolic SPHK1 and nuclear HIF1A in JAR cells under control (21% O_2_) and hypoxic conditions (1% O_2_ and 8% O_2_)- time course. β-Actin blots are shown as loading controls, n = 4 independent experiments.

SPHK1 is known to be up-regulated in many human solid hypoxic tumors [19] and has been suggested to promote tumor vasculogenesis and metastasis. As hypoxia is a key feature of early placentation, we tested whether hypoxia induces sphingosine kinase expression in two placental models. First, we tested the effect of different oxygen concentrations (8% O_2_ and 1% O_2_ versus 21% O2) on *SPHK1* mRNA and protein expression in human choriocarcinoma JAR cells, an established *in vitro* model of placental origin [33]. *SPHK1* mRNA was measured in JAR cells in a time-dependent manner, at 3h up to 16h of exposure to different oxygen concentrations. At 6h and 8h of 1% O2, *SPHK1* mRNA was increased significantly when compared to 21% O2 (Fig 1A). Western blot analysis revealed the increased cytosol abundance of SPHK1 protein at 6h, 16h and 24h upon 1% oxygen exposure (200–400% over the control (21% O2)) (Fig 1B). However, culturing the JAR cells at 8% O2, which is considered as physiological placental hypoxia, did not have any effect on SPHK1 protein during the time course (6h to 24h) when compared to 21% O2 (Fig 1C). We also evaluated the expression of hypoxia-inducible factor 1A (HIF1A) under different oxygen tensions to confirm the cells were rendered hypoxic and mounted an appropriate molecular respond. Indeed, we found nuclear protein levels of HIF1A were increased at 1% O_2_ during the time course (Fig 1B). As with SPHK1, there was no significant up-regulation at 8% O_2_ (Fig 1C).

**Fig 2:**
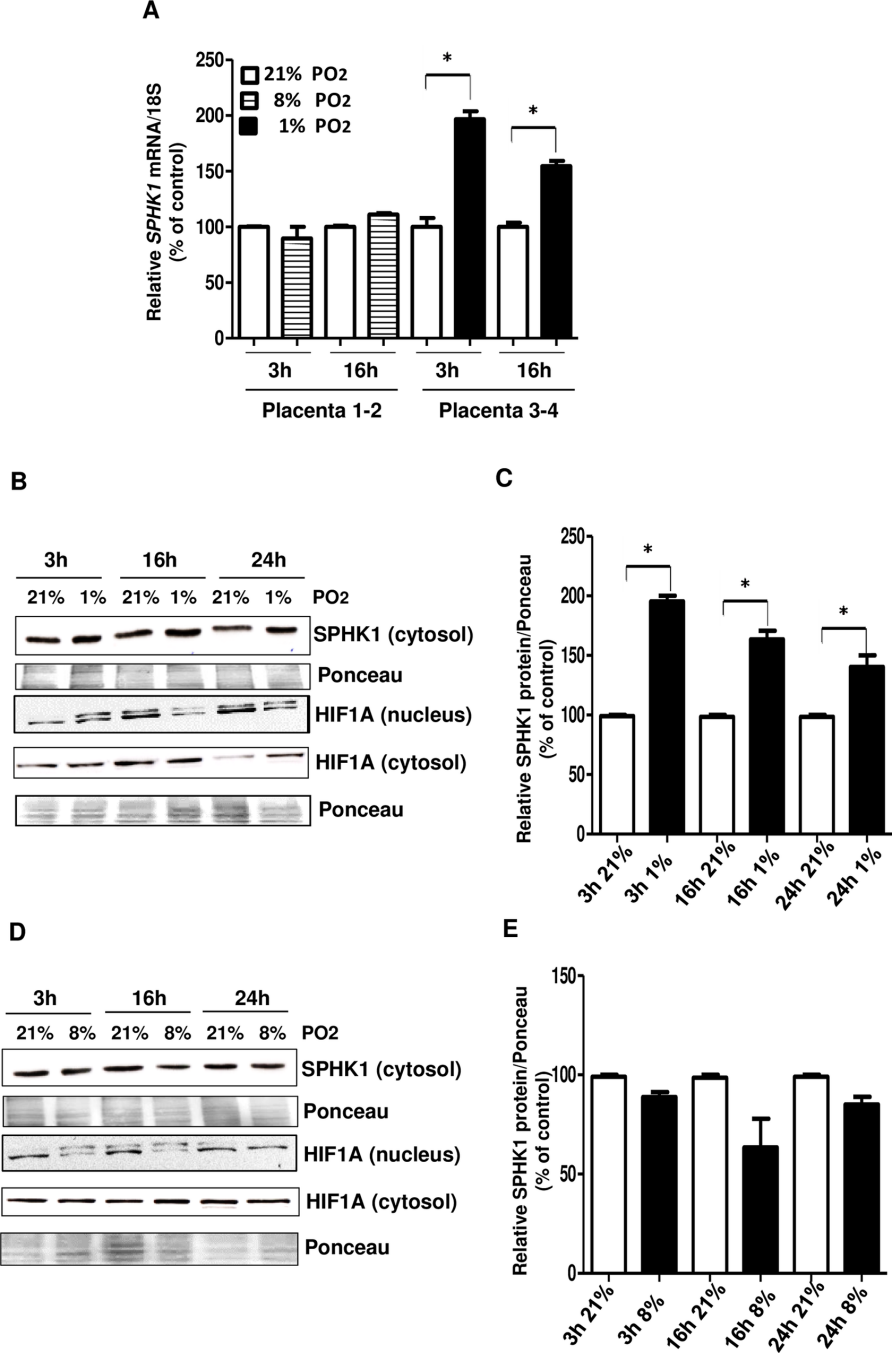
**SPHK1 levels are increased at low oxygen tension (1% PO**_**2**_**) in normal human term placental chorionic villous explants (A)** *SPHK1* mRNA relative abundance was quantified by qRT-PCR and normalized to 18S in chorionic villi exposed to hypoxia: 1% PO_2_ (dark histograms), 8% PO_2_ (dashed histograms) and 21% PO_2_ (white histograms) for 3 and 16hrs. Mean ±S.E.M, *p ≤ 0.05, n = 3. Chorionic villous explants were derived from four different normotensive patients and three independent experiments with hypoxic camera were performed. **(B, D)** SPHK1 and HIF1A protein abundance was determined by Western blot in villous explants under control (21% O_2_) and hypoxic conditions (1% and 8% O_2_) at 3, 16 and 24hrs. Ponceau stain was used as a control for uniform loading. **(C, E)** Digitized Western blots analyzed as SPHK1/Ponceau density ratios in chorionic villi. Bar graphs represent mean ±S.E.M, *p ≤ 0.05, n = 3 derived from different patients´ placental villous explants.

Next, we examined the effect of low oxygen tension on SPHK1 expression in human term villous explants from normotensive (control) patients, a well-established *ex vivo* placental model. Similarly as for the JAR cells, exposure of villous explants to 1% O2 steadily increased *SPHK1* mRNA and protein levels (at 3h, 16h and 24h) in comparison with control 21% O2-treated explants (Fig 2A and 2C). Expectedly, SPHK1 expression remained unchanged in villous explants exposed to 8% O2 as compared to control (21% O2) (Fig 2A, 2D–2E). In these experiments, we also measured the cytosolic and nuclear levels of HIF1A. Cytosolic HIF1A did not change at 1% or 8% PO_2_. Nuclear HIF1A protein in control (21% O_2_) and hypoxia-treated (1% and 8% O_2_)-villous explants appeared as a doublet on SDS-Page. Under low oxygen (1% O_2_), the early induction of SPHK1, starting already at 3h (Fig 2B) was paralleled by early activation of HIF1A in the nucleus, followed then by reduced nuclear protein levels of HIF1A with advanced time of hypoxic exposure (16h and 24h) (Fig 2B). Taking together, these results indicate that (i) hypoxic conditions up-regulate SPHK1 expression in severe hypoxia, similar to the effects seen on HIF1A, an established marker of hypoxia, which is also required for the initiation of neo-angiogenesis; and that (ii) SPHK1 might be an upstream modulator of HIF1A, and this may be facilitating neo-angiogenesis (neovascularization) in placenta.

### SPHK1 and S1PR1,-3 expression is down-regulated in placentae of preeclamptic patients

We next analyzed *SPHK1* mRNA and protein expression in human term placentae obtained from normal and preeclamptic pregnancies.

Placental tissues were obtained from 16 normal pregnancies and 17 preeclamptic pregnancies, including moderate preeclampsia (PEM) (n = 8) and severe preeclampsia (PES) (n = 9). Table 1 represents the clinical characteristics of the total group of patients used in this study. At 21–23 weeks of gestation, women who developed PE (PEM & PES) showed a significantly higher systolic and diastolic arterial pressure (p = 0.0003 and p = 0.00001, respectively) as compared to control subjects. There were no significant differences in maternal age at delivery between control and preeclamptic patients, but gestational age at delivery was significantly different between normotensive and PEM patients (p = 0.0045) and normotensive versus PES patients (p = 0.008). Additionally there was significant difference in fetus weight between normotensive and PES group (p = 0.02).

**Fig 3:**
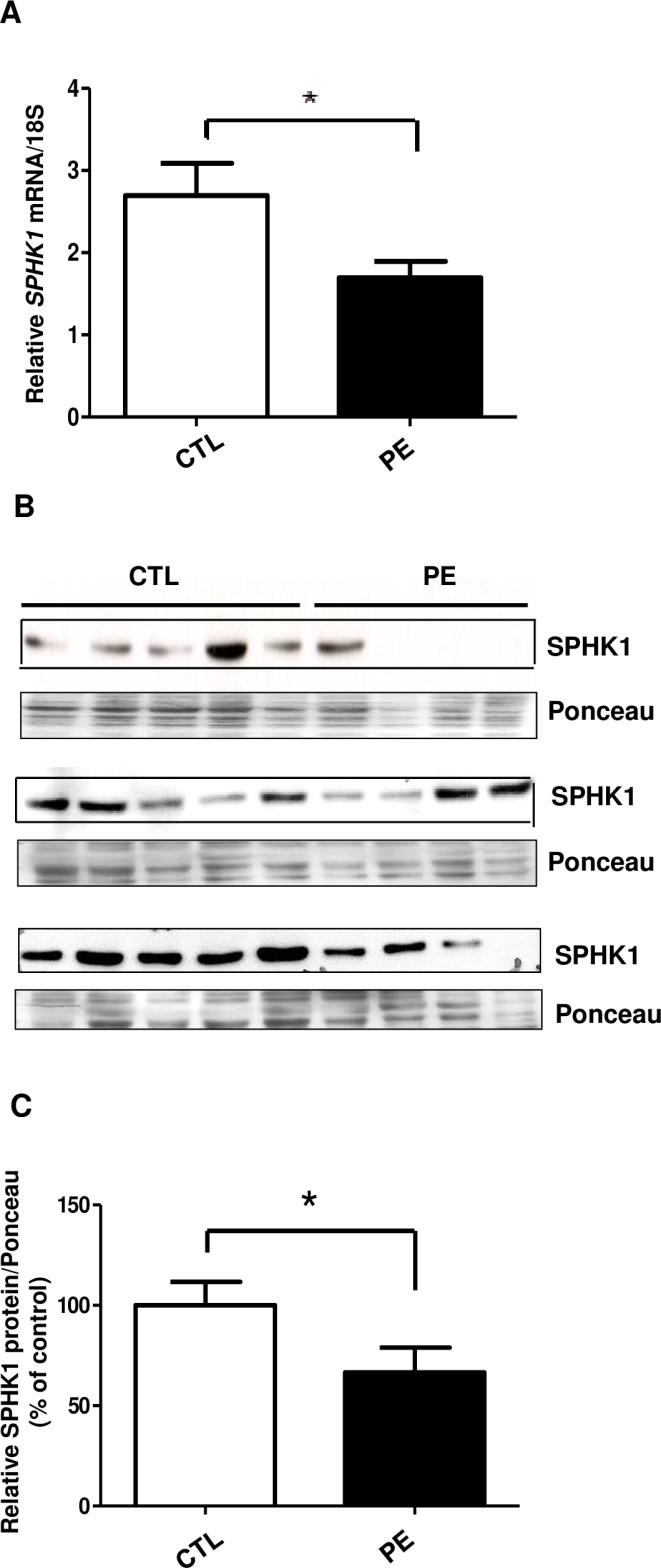
*SPHK1* mRNA and protein levels are down-regulated in term human placentae of preeclamptic patients. **(A)** qRT-PCR: bar graph represents relative *SPHK1* gene expression normalized to 18S in CTL (n = 16) and PE (n = 17) subjects **(B)** SPHK1 protein expression from control (CTL) and preeclamptic (PE) placentae was determined by Western blot. Ponceau staining was used as a loading control **(C)** Bar graph represents relative SPHK1 protein abundance normalized to Ponceau stain. Mean ±S.E.M, *p ≤ 0.05, Control (CTL): n = 16, Preeclamptic (PE): n = 17.

The expression of *SPHK1* mRNA and protein was compared between placentae from normotensive and preeclamptic pregnancies. Preeclampsia was associated with significant decrease of placental *SPHK1* gene expression (p = 0.025) (Fig 3A). SPHK1 protein content was also significantly reduced in placentae of PE patients as compared to their matched controls (p = 0.026) (Fig 3B and 3C). These data indicate that PE placentae may have a defect in their sensitivity to hypoxia and to respective up-regulation of SPHK1 expression.

**Fig 4:**
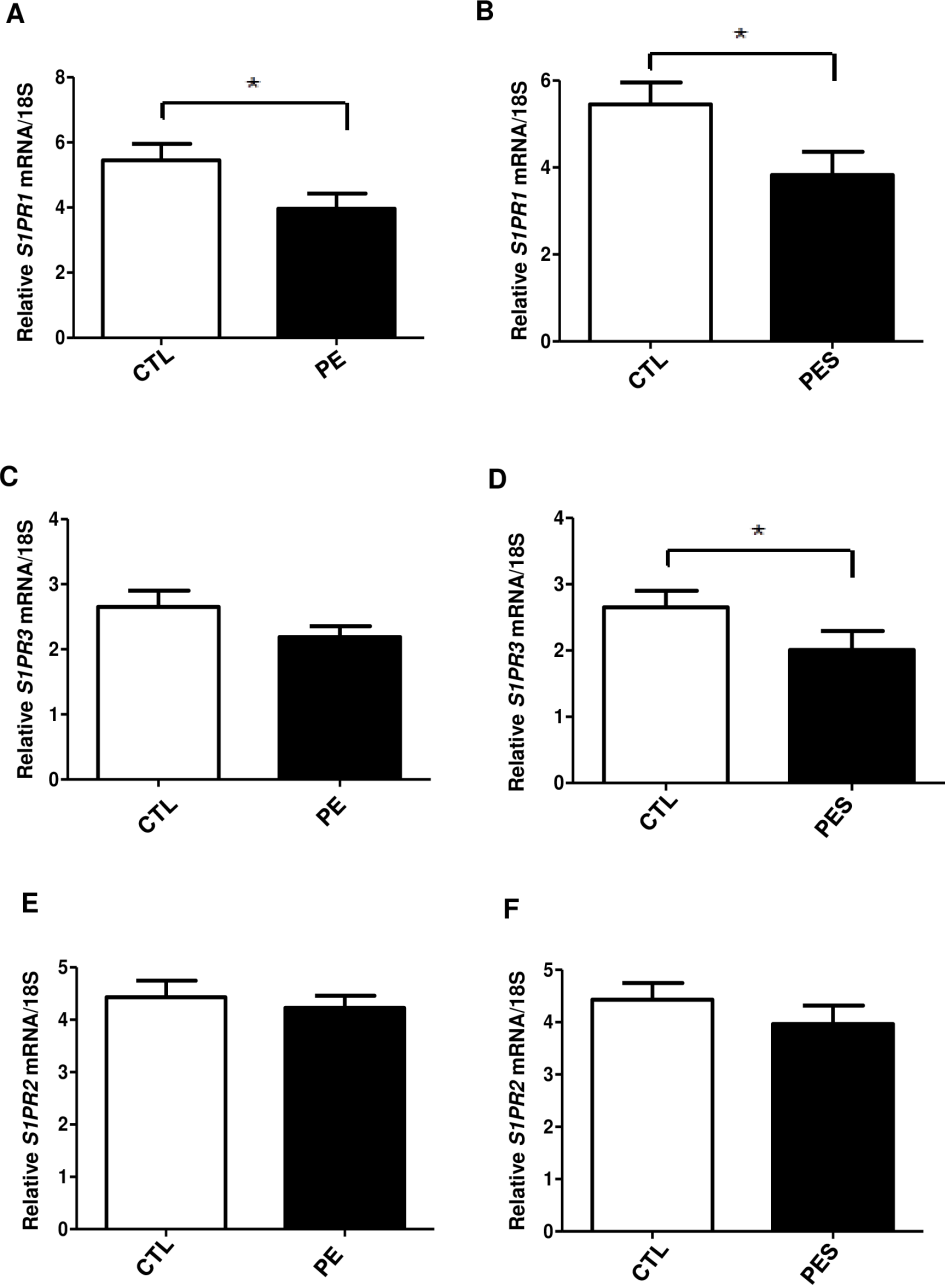
Differential expression of S1P receptors (S1PR1, -2, -3) in term placentae of preeclamptic (PE), severe preeclamptic (PES) and normotensive (CTL) patients. Real-time PCR—representative bar graphs of *S1PR1* **(A, B)**, *S1PR3* **(C, D)** and *S1PR2* **(E, F)** mRNA expression normalized to 18S in CTL (n = 16), PE (n = 17) and PES (n = 9) subjects. Mean ±S.E.M, *p ≤ 0.05, Control (CTL): n = 16 versus Preeclamptic (PE): n = 17 and CTL (n = 16) versus PES (n = 9).

Further we analyzed the expression of sphingosine-1 phosphate receptors (S1PR1, -2, -3). Significant reduction of angiogenic *S1PR1* and *S1PR3* mRNA was observed in PE and/or PES (severe PE) placental samples when compared to control group, (p = 0.025 and p = 0.043, respectively), (Fig 4A–4D). RNA levels of anti-angiogenic *S1PR2* did not differ significantly between PE and control placentae (Fig 4E and 4F). Collectively these data provide further support to the notion that SPHK1 and angiogenic S1PR1 and -3 receptors pathway is impaired in term human placentae from PE patients and might be responsible for impaired vasculogenesis/angiogenesis observed during preeclampsia.

### SPHK1/S1P receptors pathway is impaired in chorionic villi of PE patients

**Fig 5:**
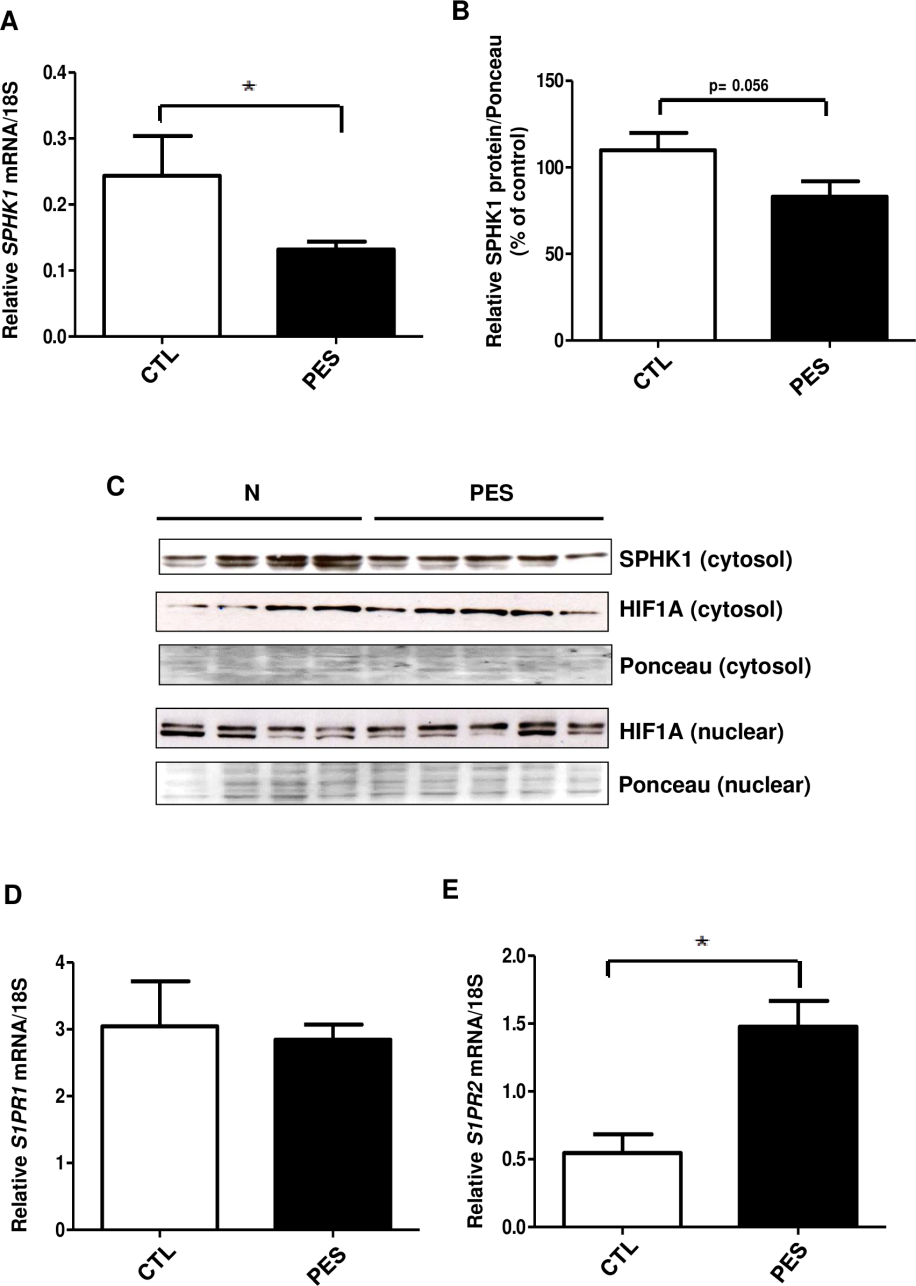
SPHK1-S1P receptors pathway is impaired in chorionic villous tissue of severe preeclampsia (PES) The chorionic villous explants were dissected from three normotensive and two severe preeclampsia placentae. For each placenta, chorionic placental trees were isolated from 4–5 different placental regions and further processed to obtain RNA and proteins. **(A)** qRT-PCR: bar graph represents relative *SPHK1* mRNA levels normalized to 18S in chorionic villi of control (CTL) and severe preeclampsia (PES) subjects. Mean ±S.E.M, *p ≤ 0.05 **(B)** Bar diagram represents relative SPHK1 protein levels in CTL and PES villous tissue. **(C)** Representative Western blots of SPHK1 and HIF1A protein in CTL and PES placental villi speicemen. Ponceau stain was used to normalize protein levels. **(D, E)** qRT-PCR: bar graphs represent relative *S1PR1* and *S1PR2* mRNA levels in control and PES chorionic villi. Mean ±S.E.M, *p ≤ 0.05

We further examined expression of the SPHK1/S1P pathway in chorionic villous placental explants isolated from PE placentae as compared to normotensive controls. The chorionic villous explants were dissected from three normotensive and two severe preeclampsia (PES) placentae. For each placenta, three chorionic placental trees were obtained from four to five different regions and further processed to isolate RNA or proteins. These analysis showed that *SPHK1* mRNA was significantly reduced in chorionic villi of PES patients (p = 0.024) (Fig 5A). In addition, we observed that expression levels of *SPHK1* mRNA in chorionic villi were much lower than in whole placenta, which may suggest that the main source of placental SPHK1 are blood erythrocytes contaminating the placental tissue. SPHK1 protein content was also significantly decreased in PES chorionic villous explants when compared to control normotensive group (p = 0.05) (Fig 5B and 5C).

The expression pattern of sphingosine-1 phosphate receptors in chorionic villi tissue was different that one observed in whole placentae, pointing to the eventual tissue specificity. In placental chorionic villi we could not detect *S1PR3* mRNA, which was indicative that this receptor is rather expressed in placental tissue of maternal origin. *S1PR1* mRNA expression did not differ significantly between PES and control chorionic villous explants (p = 0.6) (Fig 5D). However, opposite to whole placental tissue, significant up-regulation of *S1PR2* mRNA was observed in PES chorionic villi samples when compared to control group (p = 0.0087) (Fig 5E). These results may suggest that up-regulation of anti-angiogenic S1PR2 in PES chorionic villous explants might be indicative of endothelial inflammation. Hence, activation of S1PR2 signaling in endothelial cells has been shown to stimulate the production of pro-inflammatory cytokines and lipid mediators thus leading to endothelial senescence and atherogenic stimuli-induced endothelial activation [34].

Interestingly, in these analysis regarding villous explants, the expression of HIF1A protein did not follow the changes in SPHK1 expression pattern, likely because of not optimal time-point for the analysis; in *in vitro* experiments with hypoxia, the effects on HIF1A were transient and limited to early time points.

## Discussion

Bioactive sphingolipids and their synthetizing/metabolizing enzymes are key cellular mediators of many physiological processes, while imbalance of sphingolipids´ rheostat is a prerequisite to develop pathological states, such as, cancer, atherosclerosis, inflammatory diseases, to name few [22].

In the present study, we demonstrate for the first time that the SPHK1/S1P receptor pathway is impaired in placentae of preeclamptic patients and in term placental villous explants of severe preeclampsia when compared to controls. The down-regulation of placental angiogenic SPHK1 and S1P receptors (S1PR1, -3) and simultaneous up-regulation of anti-angiogenic S1PR2 receptor in placental villi point to their possible role in impaired vasculogenesis/angiogenesis observed during preeclampsia. By using *in vitro* and *ex vivo* placental models of hypoxia, we demonstrate that pro-survival and angiogenic SPHK1 is an oxygen sensing lipid enzyme, being strongly up-regulated at very low oxygen tension (1% O2) in either JAR choriocarcinoma cells or term placental villous explants. More interestingly, physiological hypoxia (8% O2) had no effect on SPHK1 expression when compared to 21% O2 control conditions. These results may suggest that SPHK1 and its product, sphingosine-1-phosphate might be an important angiogenic mediators during early placentation process, which is severely affected in PE.

Previous reports have shown that S1P and its signaling pathway is tightly regulated throughout gestation [12, 35–36]. Indeed, during normal mouse pregnancy (day 7.5pc mice decidua), angiogenic SPHK1 is significantly up-regulated (10-fold) comparing to the non-pregnant uterus [12]. Also the expression of three potent S1P receptors (S1PR1, -2 and -3) is increased in the mouse uterine decidua from 4.5 to 7.5 dop (day of pregnancy), a period of time in which extensive angiogenesis is observed in the uterus [35]. Additionally, in unilaterally pregnant ovine model, the expression levels of SPHK1 and S1PR1 are much higher in the gravid compared to non-gravid uterine horn, pointing to the important role of SPHK1-S1P pathway during placentation and embryogenesis [36]. The impairment of SPHK-S1P receptor axis has been shown to lead to vascular defects during embryogenesis. Elegant and pioneering studies performed by Proira´ and Pru´s groups have shown the impaired uterine decidualization and uterine angiogenesis in SPHK double knockout-mice (*sphk1-/-*, *sphk2-/+)* [12] and lack of microvascular expression of S1PR1 and S1PR2 receptors in oil-induced deciduomas of murine pseudopregnancy model [35].

Based on the literature, we hypothesized that the SPHK1-S1P pathway, previously well characterized angiogenic pathway involved in tumorigenesis and metastasis [19] is down-regulated in pathological state such as preeclampsia, which is compromised by insufficient endometrial/placental vascularization. Indeed, our data demonstrated the reduction of *SPHK1* mRNA and protein levels in term preeclamptic placentae and as well chorionic villous explants of severe preeclampsia when compared to the control subjects, confirming that SPHK1 is expressed not only by the whole placenta, but also fetal tissues. However, it needs to be mentioned that levels of *SPHK1* mRNA were detectable, but significantly lower in chorionic villi when compared to whole placental tissue, which indicates tissue specificity in SPHK1 expression pattern. Notably, our findings are in line with the recently published results from Melland-Smith et al. where they observed the reduced levels of circulating angiogenic S1P in serum of preeclamptic patients in comparison with the control subjects [37]. Moreover, in the same PE patients the levels of placental and serum ceramides (Cer16, Cer18, Cer20, Cer24) were significantly elevated as compared to those found in control normotensive women, suggesting the imbalance of sphingolipids rheostat during preeclampsia [37].

Indeed, sphingosine kinase (SPHK) has been shown to be critical for the maintenance of physiological sphingolipids´ balance, as it not only produces the pro-growth, anti-apoptotic bioactive lipid S1P, but also decreases levels of pro-apoptotic ceramide (Cer) and sphingosine (Sph) [13]. In fact, in addition to the above described findings in preeclampsia [37], the recent reports in human IUGR clearly showed that down-regulation of SPHK1 is accompanied by increased placental levels of sphingosine (Sph), which is a substrate for sphingosine kinase [38]. In animal model, mice SPHK-double KO (*sphk1-/-*, *sphk2-/+)*, SPHK deficiency led to enormous accumulation of dihydrosphingosine and sphingosine and elevation of ceramides in pregnant mice uteri [12].

The causes of down-regulation of SPHK1 observed during preeclampsia are unknown. One limitation of our study is that we evaluated term placentae, but would be of great interest to determine SPHK1 expression pattern in 1^st^ trimester human placentae in patients at risk of developing preeclampsia by using intrauterine artery Doppler as a predictive factor. It would be tempting to speculate that in PE there is a defect in SPHK1 expression either on maternal or paternal side already before preconception or that observed decrease of SPHK1 is secondary appearing in the first weeks of gestation and is regulated by unidentified yet factors and/or cellular and physiological events that leads to impaired trophoblast invasion and inadequate artery remodeling.

The hypothesis that in preeclampsia SPHK1 pathway can be down-regulated in the early phases of placentation process is supported by our *in vitro* and *ex vivo* placental models of hypoxia, (JAR cells and term placental explants), in which we observed strong up-regulation of SPHK1 in the presence of very low oxygen content (1% O_2_), but not at the physiological hypoxia (8% O_2_). Augmentation of SPHK1 has been widely reported in many cancer cells, as it serves as a hallmark of angiogenic properties of the tumors [20]. Indeed, Ader et al proposed that SPHK1 can act as a master regulator of hypoxia, by stabilizing and activating HIF1A [19]. Undoubtedly SPHK1 and HIF1A are the part of the same canonical pathway during hypoxia. As hypoxia is a hallmark of the early weeks of gestation, it would be attractive to hypothesize that SPHK1 is first activated and acts upstream of HIF1A to stabilize its accumulation, but then as SPHK1 is continued to be overexpressed it might then down-regulate HIF1A. In preeclampsia, it is possible that decreased SPHK1 levels lead to persistent HIF1A stabilization and activity. Thus, in turn, might continue to propagate the placental disease process [3]. In further support for crosstalk between SPHK1 and HIF1A is recent data demonstrating that exposure of choriocarcinoma JEG3 cells to TGFβ3, a downstream target of HIF1A and negative regulator of trophoblast invasiveness, strongly reduces the levels of SPHK1 protein [38].

The discrepancy in our results found between *ex vivo* placental model (reduced SPHK1 in PE versus normal placentae) and *in vitro/ex vivo* placental models of hypoxia (increased SPHK1 in JAR cells and villous explants of normal term placentae at 1% O_2_) may be explained by the fact that PE placentae may have an aberrant response to hypoxia and do not up-regulate SPHK1 expression. As noted above, such a response may then be expected to result in persistent HIF1A activation, propagating the placental preeclamptic disease process. If this is the case, identifying the molecular brake that represses increased SPHK1 expression in the chronically hypoxic preeclamptic placentae may in fact reveal a potential therapeutic target(s).

The consequences of blocking SPHK1-S1P pathway *in vitro* have been shown to have negative impact on angiogenesis, cell proliferation and migration [36, 39]; the physiological and cellular processes being impaired in PE. For example, treatment of human and ovine endothelial cells with FTY720, a S1P mimetic, has been reported to block the cell invasion in an *in vitro* model of sprouting angiogenesis [36]. In addition, pharmacological or siRNA inhibition of SPHK led to suppressed proliferation of glioma cells under hypoxia [39]. Interesting study done on adult mouse cardiomyocytes null for the SPHK1 gene, showed that these cells were more vulnerable to stress of hypoxia and that exogenous S1P was able to rescue them from hypoxic cell death [40].

Our results with expression pattern of S1P receptors (S1PR1,-2,-3) displayed tissue specificity with S1PR1 and S1PR3 being significantly decreased in PE human term placentae and S1PR2 being increased in chorionic villous tissue of severe preeclampsia. Although S1PR1 and S1PR3 are expressed in both embryonic and uterine tissues [35, 41], we were unable to detect significant levels of *S1PR3* at the mRNA level in chorionic villi. More interestingly the expression pattern of S1P receptors observed by us in human placentae and villous explants of preeclamptic patients is opposite to that detected in cancer cell lines, pointing to impaired angiogenesis during preeclampsia in contrast to enhanced angiogenesis during tumorigenesis [28].

As we mentioned before, S1P is considered as an important regulator of vascular formation, barrier protection and vascular tone by acting on its G-protein coupled receptors, particularly via its angiogenic receptors; S1PR1 and S1PR3 [28]. In our study, these two receptors were significantly decreased in placental tissue of preeclamptic subjects when compared with controls. [35]. In fact, the severity of lacking these receptors has been reported in knockout murine models. Deletion of S1PR1 has been shown to be lethal to the mouse embryo due to hemorrhaging [29]. Moreover, the double-knockout embryos (*S1PR1*^-^/*S1PR2*^-^) or (*S1PR1*^-^/*S1PR3*^-^) have been reported to exhibit more severe vascular maturation defects and earlier intrauterine death when compared with S1PR1-null phenotype alone [42]. On the other hand, our results with increased *S1PR2* mRNA levels in chorionic villi of PES patients point to anti-angiogenic properties of S1PR2. Interestingly, endothelial S1PR2 signaling plays a key role in mediating endothelial inflammation [34]. Levels of S1PR2 are markedly increased in senescent, atherosclerotic and inflamed endothelia [43, 44]. In EC cells, S1PR2 mediates S1P-induced inhibition of cell migration and capillary-like tube formation, pointing to its inhibitory role in angiogenesis [45].

Results of the present study demonstrate impairment of SPHK1-S1P receptors pathway in human placentae and chorionic villous explants of PE patients, suggesting that it might be involved in challenged angiogenesis observed in preeclampsia. Moreover, *in vitro* and *ex vivo* studies clearly show up-regulation of angiogenic SPHK1 under low oxygen tension in contrast to lack of SPHK1 stimulation under the conditions of placental physiological hypoxia. This may indicate that SPHK1 and its product, sphingosine-1 phosphate might be an important angiogenic mediators during early placentation process, which is severely affected in preeclampsia. This study will enhance our understanding of the complex molecular network underlying pregnancy-associated disorder—preeclampsia, and more importantly in the future, may lead to discovery of potential sphingolipid-related biomarkers of this disease and thus to development of potential drugs directed to sphingolipids or sphingolipids-related intracellular or extracellular targets.
